# Case report: A case of incontinentia pigmenti

**DOI:** 10.3389/fmed.2023.1164394

**Published:** 2023-05-12

**Authors:** Lingfeng Xie, Yong Zhu, Liya He, Bing Yu, Jiajue Wang, Ruiqiang Fan, Xiumei Mo, Yu Zhang, Ting Xie

**Affiliations:** ^1^The Second Clinical Medical College, Guangzhou University of Traditional Chinese Medicine, Guangzhou, China; ^2^Department of Dermatology, The Second Affiliated Hospital of Guangzhou University of Chinese Medicine (Guangdong Provincial Hospital of Chinese Medicine), Guangzhou, China; ^3^Guangdong Provincial Institute of Chinese Medicine Dermatology, Guangzhou, China; ^4^Department of Pathology, The Second Affiliated Hospital of Guangzhou University of Chinese Medicine (Guangdong Provincial Hospital of Chinese Medicine), Guangzhou, China

**Keywords:** incontinentia pigmenti, ectodermal dysplasia, histopathological examination, IKBKG/NEMO mutations, female infant

## Abstract

Incontinentia pigmenti (IP) is a rare neuroectodermal dysplasia caused by mutations in the IKBKG gene. We present a case of a 4-month-old female infant with erythematous vesicular skin lesions on the trunk and extremities. Histopathologic examination of the blisters revealed an eosinophilic infiltrate. Further investigation revealed that her mother had three unexplained miscarriages and two normal uncomplicated pregnancies, resulting in the birth of two male infants. We performed a comprehensive genetic evaluation to rule out the interference of pseudogene IKBKGP, and the infant was finally diagnosed with IP. During the subsequent 2-year follow-up, we observed a significant improvement in her dermatologic symptoms, with no evidence of recurrence, and there were no other associated symptoms in the hair, nails, oral mucosa, eyes, or central nervous system.

## Introduction

Incontinentia pigmenti (IP, Bloch–Siemens Syndrome, OMIM 308300) is an X-linked hereditary skin condition with an estimated incidence of 1.2 per 100,000 (http://www.orpha.net/orphacom/cahiers/docs/GB/Prevalence_of_rare_diseases_by_alphabetical_list.pdf) (1, 2). This condition is usually fatal in male fetuses and manifests itself in female fetuses as a variety of abnormalities of the skin, hair, nails, teeth, eyes, and central nervous system. The most common manifestation is cutaneous, which occurs in Blaschko's lines and is characterized by four classic stages: inflammatory vesicles, verrucous plaques, a distinct pattern of hyperpigmentation, and dermal scarring. IP is typically diagnosed by tissue biopsy and skin changes; however, due to the fact that it is caused by mutations in the IkBKG (inhibitor of nuclear factor kappa-B kinase subunit gamma)/NEMO (nuclear factor kappa-B (NF-κB) essential modulator) genes, genetic testing is becoming increasingly popular as an effective method of diagnosis (1).

## Case report

**Figure 1:**
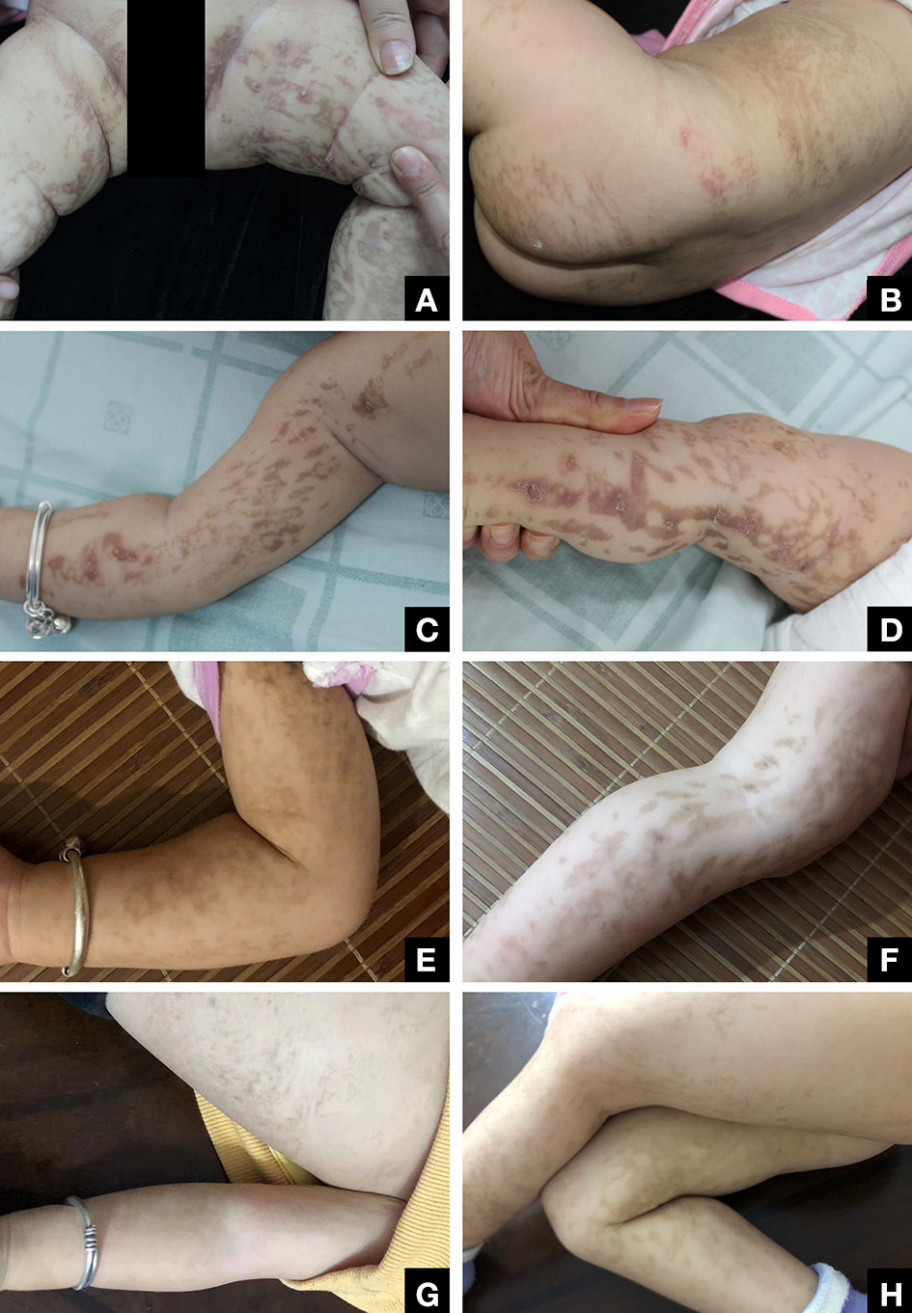
**(A, B)** Patient at 4 months of age with inflammatory blisters with Blaschko's lines on the extremities. **(C, D)** Patient at 6 months of age with warty papules and hyperpigmentation. **(E, F)** At 19 months of age, the patient has few residual verrucous growths on the extremities, and the residual pigmentation is gradually fading. **(G, H)** Skin symptoms largely resolved by the time the patient was 2 years old.

We present the case of a female infant born at term of a non-consanguineous marriage who presented to our hospital at 4 months of age with cutaneous eruptions covering her body, which had first appeared when she was 5 days old. The eruptions, consisting of erythematous papules and vesicles, spread to her extremities and trunk in a pattern consistent with Blaschko's lines. By 6 months of age, the initial blistering lesions had resolved, but the typical IP skin changes had progressed to the next stage, manifesting as warty plaques and hyperpigmentation in the same location as the initial eruptions, accompanied by mild pruritus (Figure 1).

According to the patient's mother, the patient was intellectually alert and had no history of seizures from birth until the time of examination, and there were no significant ocular anomalies observed. Due to the patient's young age and inability to follow commands, the parents opted not to perform further ocular and central nervous system testing. A thorough physical examination revealed that the scalp, hair, nails, and oral mucosa were all normal.

During the consultation, we obtained a detailed medical history and learned that the patient's mother had had three unexplained miscarriages, all resulting in the stillbirth of male fetuses. The first pregnancy ended in a stillbirth at 23 weeks, with amniocentesis revealing cloudy amniotic fluid. However, no relevant screening for chromosomal aberrations was performed, as the parents did not take the matter seriously. In the two subsequent pregnancies, nuchal translucency thickening was detected during prenatal screening, and hydrops fetalis was observed by ultrasound examination at 12 weeks gestation. Furthermore, according to the patient's mother, her own mother had also experienced an unexplained miscarriage of a male stillborn. No effect of the condition was reported to have been observed in the patient's father or any other members of his immediate or extended family. In addition, the patient had two brothers, a 9-year-old and a 4-year-old, both of whom were reportedly free of any abnormalities.

**Figure 2:**
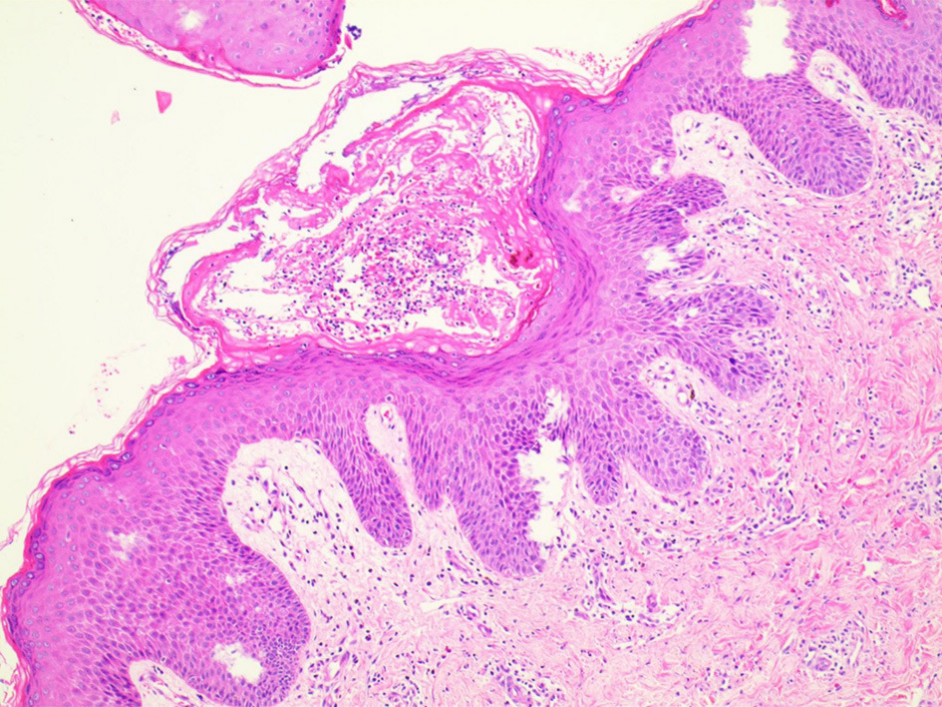
Microscopic examination showed eosinophils infiltrating the dermis and vesicles.

**Figure 3:**
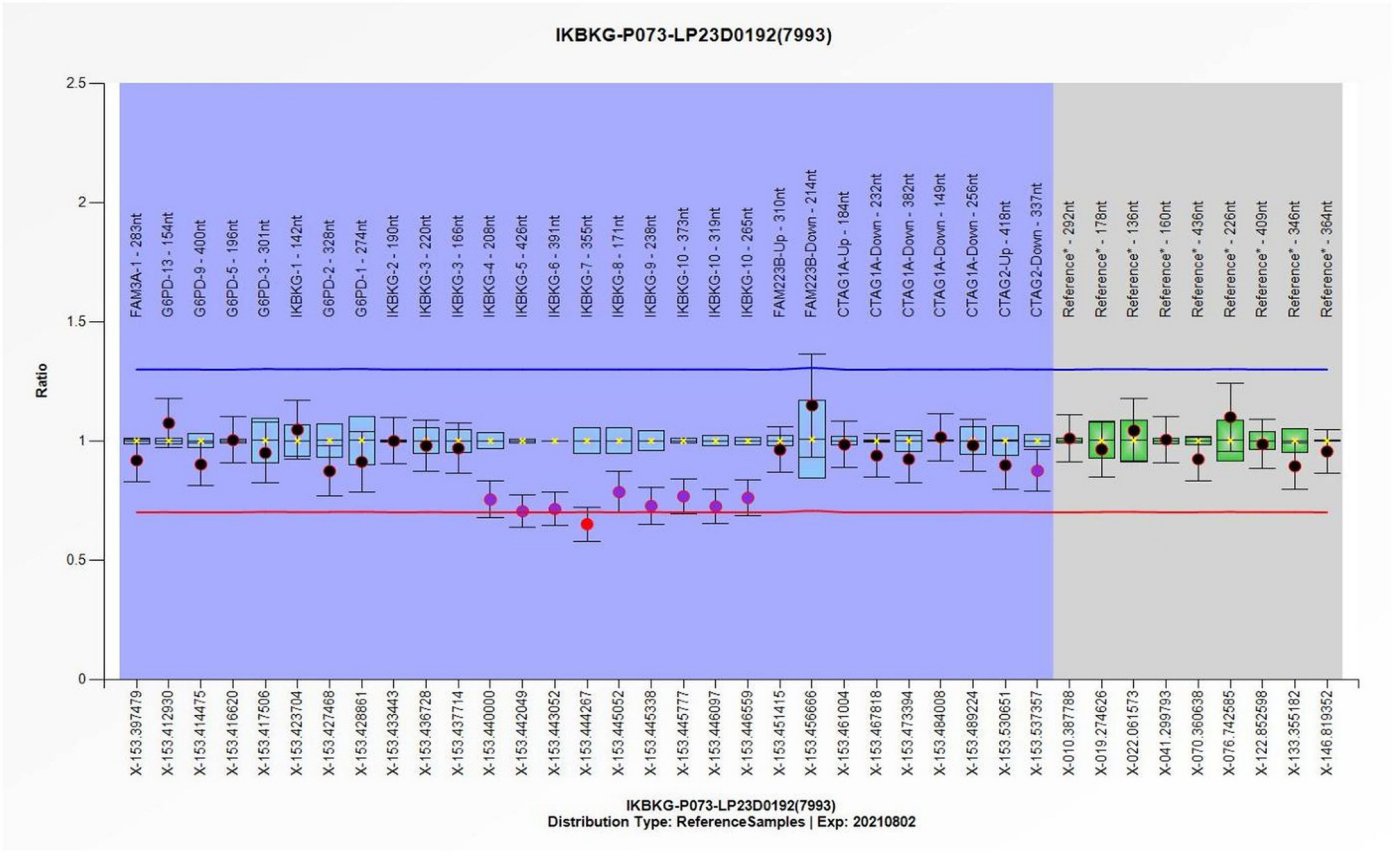
The individual exons of the patient's IKBKG gene were detected using the multiple ligation-dependent probe amplification (MLPA), with the DNA from normal individuals serving as a reference, thereby enabling the identification of deletions and duplication mutations in the gene. Quantitative MLPA plots show the individual exons on the horizontal axis and the ratio to normal female subjects on the vertical axis.

**Figure 4:**
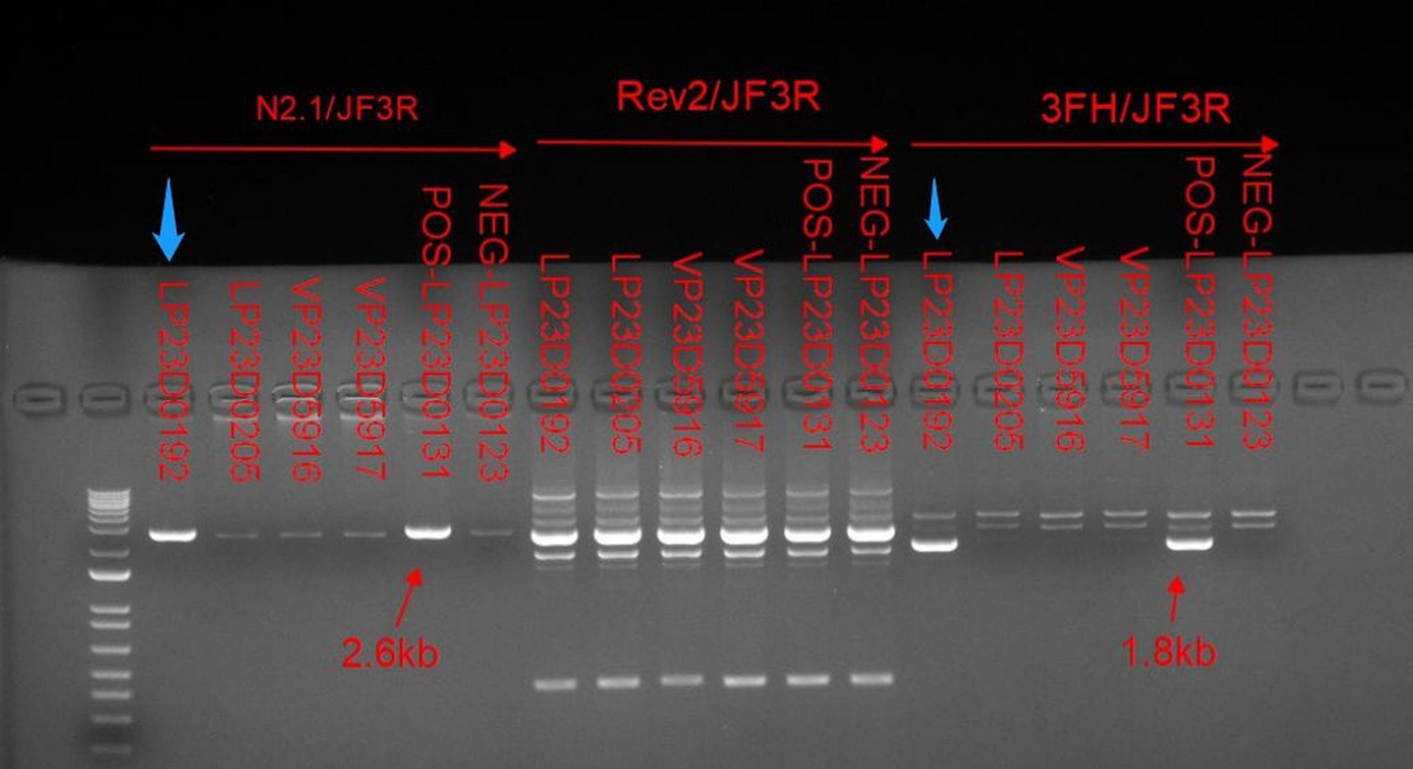
Relative to the positive control indicated by the red arrow. Long PCR with N2.1/JF3R and 3FH/JF3R primers revealed IP-specific 2.6-kb and 1.8-kb bands in patient samples, as indicated by the blue arrows. Conversely, the bands amplified with the IKBKGP-specific primer (JF3R/Rev2) showed no discernible specificity between patients and controls.

In light of the specific cutaneous clinical presentation, we performed a skin biopsy, which revealed subtle spongiosis with scattered eosinophilic infiltration in the lower epidermis and at the dermal/epidermal junction (Figure 2). Additionally, mild dermal infiltration of eosinophils and lymphocytes was also observed, in addition to vesicles with eosinophilic cells and hyaline cytoplasm surrounded by scattered dyskeratotic keratinocytes within the epidermis. Immunofluorescence staining results for C3, IgA, IgG, and IgM were all negative. Toluidine blue staining was negative, and immunohistochemical staining was negative. This distinctive skin biopsy presentation is different from the microscopic presentation of epidermolysis bullosa. For further diagnosis, we tested the patient for the IP IKBKG gene; however, no pathogenic variants in the exon coding region of the IKBKG gene were detected by Sanger sequencing (data not shown). Subsequently, we used multiple ligation-dependent probe amplification (MLPA) (P073-A1; MRC-Holland, Amsterdam, The Netherlands), which revealed a deletion mutation in exons 4–10 of IKBKG (Figure 3) and performed a long PCR to verify the positive results of MLPA and to eliminate the potential interference of pseudogenes (Figure 4) (3).

The diagnosis of IP was established based on the patient's skin lesions, biopsy results, family history (three miscarriages), and the presence of deletion mutations in IKBKG (4). To manage the patient's skin symptomology, a combination of oral antihistamines and topical steroid cream was prescribed. During the 2-year follow-up, the patient's milk teeth eruption was reported to be normal, and no anomalies were observed in the hair, nails, or breasts. The patient did not exhibit any abnormal manifestations of vision or central nervous system, such as seizures or learning deficits, as reported by the patient's parents. Nonetheless, we recommended conducting appropriate imaging studies as a precautionary measure (5). The patient's skin condition continued to improve during this period, and by the age of 2 years, the skin discoloration had substantially subsided (Figure 1).

## Discussion

Incontinentia pigmenti (IP) is an extremely rare X-linked dominant genodermatosis that affects a variety of ectodermal tissues, resulting in skin, teeth, hair, and nail abnormalities. The cutaneous eruption is an almost universal symptom and is often the most noticeable. IP is caused by mutations in IKBKG, which encodes the gamma regulatory subunit of the inhibitor of nuclear factor kappa-B kinase, a necessary component for the activation of the NF-κB pathway (6). NF-κB is responsible for the expression of genes that control cell development, immune and stress responses, inflammation, and protection against TNF-induced apoptosis. When IKBKG is not expressed correctly, cells become hypersensitive to apoptotic signals, leading to the development of various inflammatory responses that are particularly active in ectodermal cells (2).

Deletion mutations caused by intrachromosomal rearrangements deleting exons 4–10 of IKBKG account for 90% of IP mutations, while a highly homologous pseudogene IKBKGP exists distal to the IKBKG in a tail-to-tail direction (2, 7). The sequence of exons 3–10 of IKBKG has 99% homology to the corresponding region of IKBKGP. Which contains introns resulting in difficulties in the analysis of IKBKG mutations (8). Since the presence of IKBKGP interferes with the detection in IKBKG, specific methods are required to distinguish the true mutations. Long PCR with IKBKG-specific primers, N2.1 and 3FH, and IKBKGP-specific primer, Rev2, were used to verify that the deletion of exons 4–10 occurs in IKBKG but not in IKBKGP (Figure 4). Only in the case of IP, amplification between the forward primers, 3FH and N2, which are located upstream of exon 3, and the reverse primer, JF3R, which is located in the rearranged EcoRI fragment, produces bands of 1.8 and 2.6 kb, respectively (2, 3). In parallel, no specific bands were amplified between the IKBKGP-specific primer and JF3R, indicating that deleterious mutations occurred in IKBKG.

Although the patient's parents ultimately decided not to test other family members, they provided us with detailed information about their family history. The patient's father had no IP-related symptoms as a child, and his family had no history of miscarriages. We suspect that the mutated X chromosome was passed down through the mother's family, which would explain why the patient's grandmother had an unexplained miscarriage. This further validates our IP diagnosis. Although the classic IP phenotype is almost exclusively female, germ cell mosaicism has been reported, which allows mutations to be passed from father to daughter (9). The patient had no other sisters for us to observe, and the cause of the miscarriage in her mother's family is unknown. Genetic testing of the patient's entire family is necessary to fully and completely understand the genetic tree of IP patients. Regrettably, the patient's family was unable to complete this examination due to economic constraints.

IP has a female-to-male ratio of 37:1 because it is generally fatal to males, but inactivation of the mutant X chromosome allows females to survive (10). The patient's mother's three unexplained miscarriages were strongly suspected to be caused by lethal damage to the male fetus caused by the mutated gene, while the patient's two unaffected brothers inherited a healthy X chromosome. In female infants, a proportion of mutated chromosomes that escape inactivation causes a variety of clinical manifestations; as they grow older, the proportion of non-mutated cells increases in a process known as skewed X inactivation, and the skin symptoms gradually resolve (7). By 19 months of age, the patient's patchy wartlike growths had almost entirely resolved, and the remaining hyperpigmentation had gradually faded (Figure 1). This outcome is consistent with the findings reported in the existing literature.

The distribution of lesions based on Blaschko's lines in patients with IP is a remarkably accurate representation of the developmental pathway of ectodermal tissue (11). Impairment of the vascular modeling and hair follicle induction involved in the NF-κB signaling pathway can affect organs associated with ectodermal development, such as the hair, teeth, central nervous system, and eye, resulting in a variety of clinical manifestations (7). In the case of our patient, severe cutaneous symptoms were observed in multiple areas of the limbs and trunk, while minimal involvement of other organs was noted. Whether there is a correlation between the severity of skin symptoms and the severity of other manifestations, and whether the degree of skewed inactivation of mutant X chromosomes in different organs and the proportion of inactivation escape potentially influence this correlation remains to be investigated.

The patient's mother had a history of three miscarriages: one stillborn fetus and two cases of NT thickening and hydrops fetalis. However, due to financial constraints and lack of attention, genetic testing of the amniotic fluid was not performed by the patient's family. NT thickening is typically a screening test for chromosomal anomalies, but it may also indicate other factors, such as congenital heart disease, genetic syndromes, or neurodevelopmental delay. In the study by Bilardo, children with neurodevelopmental delay, a characteristic manifestation of IP, had NT measurements >4 mm during gestational testing (12). Therefore, we suggest that fetuses who are unable to undergo genetic testing should consider an NT measurement >4 mm combined with recurrent fetal edema as a potential indicator of IP. This may help to increase parental concern and prompt further evaluation.

From a clinical standpoint, it is imperative to distinguish IP from other dermatologic conditions, such as mastocytosis and hereditary epidermolysis bullosa. To confirm the diagnosis, a pathologic biopsy can be used in conjunction with genetic testing as an expedient and reliable method.

Cells containing the mutated gene may remain in the body after the lesions have healed, suggesting that various clinical factors may cause IP to recur (8). Any observed abnormalities, including skin findings, central nervous system findings, and ophthalmologic findings, should be evaluated as soon as possible as they may indicate a relapse of IP (13).

## Data availability statement

The original contributions presented in the study are included in the article/supplementary material, further inquiries can be directed to the corresponding author.

## Ethics statement

The studies involving human participants were reviewed and approved by the Medical Ethics Committee of Guangdong Provincial Hospital of Chinese Medicine. Written informed consent to participate in this study was provided by the participants' legal guardian/next of kin. Written informed consent was obtained from the minor(s)' legal guardian/next of kin for the publication of any potentially identifiable images or data included in this article.

## Author contributions

LX, TX, and XM contributed to the conception and design of the study and refined the manuscript. YZhu and BY collected and organized the data. RF and LH followed up with patient. LX wrote the first draft of the manuscript. JW processed the images in the manuscript. YZha provides support in the field of pathology. All authors contributed to the revision of the manuscript, read, and approved the submitted version.
